# Nature of a Tetrabutylammonium Chloride–Levulinic Acid Deep Eutectic Solvent

**DOI:** 10.1021/acs.iecr.3c02102

**Published:** 2023-11-15

**Authors:** Alberto Gutiérrez, Sara Rozas Azcona, Lorena Zamora Pastor, Cristina Benito, Mert Atilhan, Santiago Aparicio

**Affiliations:** † Department of Chemistry, University of Burgos, Burgos 09001, Spain; ‡ Department of Chemical and Paper Engineering, Western Michigan University, Kalamazoo, Michigan 49008-5462, United States

## Abstract

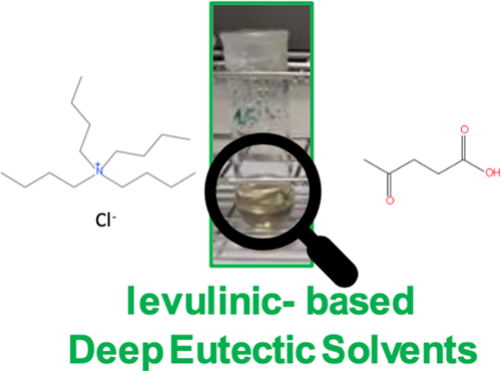

A deep eutectic solvent was formed by considering the mixtures of tetrabutylammonium chloride and levulinic acid, and it is studied via a combined theoretical and experimental approach. Physicochemical properties were measured as a function of temperature, providing a macroscopic characterization of the fluid. Quantum chemistry and classical molecular dynamics simulations were carried out for the nanoscopic characterization, providing attention to the nature, extension, and dynamics of the hydrogen bonding network, which is at the root of the properties of the fluid. The reported study allows multiscale characterization of this fluid as an archetypical example of a natural, low-cost, and sustainable fluid.

## 1 Introduction

**Figure 1:**
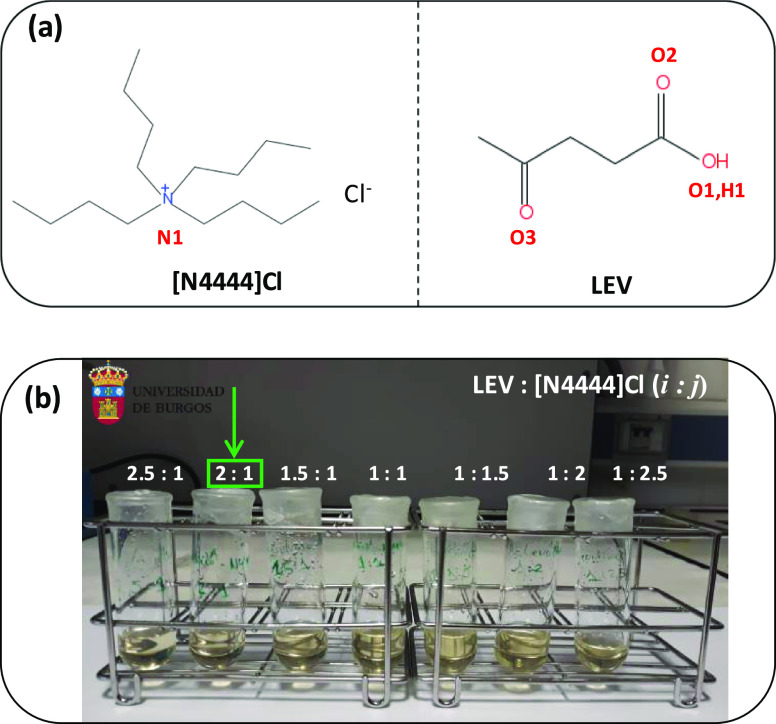
(a) Molecular structures of compounds used in this work. Atom labeling is indicated. (b) Pictures of the samples considered in this work for the LEV:[N4444]Cl at *i*:*j* mole ratios. All of the studied *i*:*j* mole ratios led to liquid DESs at 293 K (the temperature for which the pictures were obtained). A green arrow indicates 2:1 DES, for which experimental and theoretical studies were carried out.

Levulinic acid (4-oxopentanoic acid, LEV; Figure 1) is a natural compound that can be cost-effectively produced from biomass using different approaches.^1,2^ LEV has been considered to develop platforms in applications such as polymers,^3^ pharmaceutical,^4^ food industry,^2^ synthesis, and catalysis,^5^ as well as a precursor for the preparation of high-value compounds into a biorefinery approach.^6^ LEV is nontoxic (acute oral toxicity LD_50_ = 1850 mg kg^–1^)^7^ and moderately eco-toxic^8^ and presents suitable environmental properties.^9^ The safety assessment of this molecule led to consider it safe, both for human and environmental exposures,^10^ and thus it may be considered a sustainable chemistry approach to several technologies.^11,12^ Therefore, the interest in LEV-based materials and technologies has increased in these last years both in industry and academia with an expected large increase in world market demand in this decade.^13^

Among the novel applications of LEV, its use as a molecule for developing deep eutectic solvents (DESs)^14^ has been considered these last years. DESs are formed by the proper combination of Lewis and Brönsted acids and bases, leading to strong intermolecular forces, typically hydrogen bonding, which produce a large decrease in the melting temperature in comparison with those of the neat components. According to the type of components considered in the DES, they are classified as types I–V.^15−17^ Particular attention has been paid in the literature to type III DESs, which are formed by the combination of a hydrogen-bond acceptor (HBA) (e.g., quaternary ammonium salts) and a hydrogen-bond donor (HBD) (e.g., an organic acid), for which the melting temperature depression (eutectic point) is produced at certain HBA:HBD mole ratio through the development of suitable intermolecular hydrogen bonding.^18^ Type V DESs have been recently proposed by the combination of nonionic HBA and HBD components.^19^ Additional classification of DESs can be developed considering their hydrophobic or hydrophilic character,^20^ which is of relevance as water content has a large effect on DES properties^21,22^ as well as the water content requirements for different DES applications.^23^ Moreover, DESs may be classified considering the origin of DES components. In this way, DES based on components of natural origin has been proposed,^24^ leading to the so-called natural DESs (NADES),^25^ which are low cost, nontoxic, readily biodegradable, and thus a suitable platform for DES development into sustainable chemistry approach.^26^ Therefore, the research on DES has probed their suitability for a large collection of technologies,^18,27^ for which suitable DES components are needed in terms of specific requirements. Therefore, the use of LEV for DES development may be considered a suitable option both in terms of sustainability and economy, thus providing a connection of DESs with biorefinery applications. Maugeri et al.^28^ developed LEV-based DES with choline chloride (ChCl) as HBA, providing its physicochemical characterization as well as its water sorption behavior. Additional studies of ChCl: LEV DES were reported by Florindo et al.,^29^ showing a moderately viscous fluid (320.6 mPa s at 293.15 K) with a high decomposition temperature (449.72 K). LEV-based DESs considering alkylammonium chloride and bromide as HBAs were studied by Li et al.^30^ LEV in combination with amino acids and related compounds (proline betaine) were prepared by Sánchez et al.^31^ Although these compounds presented suitable thermal stability, their main problem was their large viscosity, which may hinder their application in technologies requiring suitable heat- or mass-transfer operations. Moreover, LEV-based DESs have been considered for applications such as CO_2_ and SO_2_ capture,^32−34^ cellulose treatment,^35^ separation of azeotropic mixtures,^36^ fuel dearomatization,^37^ pesticide extraction,^38^ heavy metal extractions,^39^ biomolecule solubilization,^40^ among others. Available studies on the nanoscopic characterization of LEV-based DES using molecular modeling approaches are very scarce. Classical molecular dynamics (MD) studies on ChCl-LEV were reported by Mainberger et al.^41^ and Doherty et al.^42^ and a previous study by our group,^43,44^ whereas quantum chemistry studies were also reported by our group.^45,46^ Considering the current database in the open literature, in order to design LEV-based DESs in relevant technologies, there is a need to establish solid know-how on the nanoscopic features of LEV-based DES as well as their connection with macroscopic features and physicochemical properties with emphasis on the structure–property/activity relationships. For this purpose, a type III DES based on DES through the combination of tetrabutylammonium chloride ([N4444]Cl) and LEV (Figure 1) was considered in this work as an archetype and their properties were studied using a combined experimental and computational approach to provide a full characterization of this fluid, which can be used for designing and understanding DES properties and nature. The use of molecular modeling for the study of DES has been analyzed in the literature.^47,48^ The main outcomes coming from molecular simulations are the prediction of physicochemical properties, nanoscopic description of DES properties, in particular hydrogen bonding nature, strength, and extension, and the development of reliable structure–property/activity relationships, which can be used for proper design of suitable DES for different technologies. Therefore, the use of a combined experimental and computational approach considered in this work provides a multiscale characterization of the considered DES advancing in the knowledge of the micro- and macroscopic properties of DESs.

## 2 Materials and Methods

### 2.1 Chemicals

The specifications for the chemicals used in this work are reported in Table S1 (Supporting Information). The LEV–[N4444]Cl DES was prepared by weighing (Mettler AT261 balance, ±1 × 10^–5^ g) suitable amounts of [N4444]Cl and LEV, upon mixing heating up to 313 K with stirring led to the formation of transparent (slightly yellowish) liquid phases (Figure 1) for the considered mole ratios. All of the samples remained in the liquid state after cooling to ambient temperature. Additionally, all of the considered DES were cooled to 273 K, which remained in the liquid state, thus assuring the liquid range of the studied DESs. The reason for the selection of 313 and 273 K for preparing and studying the stability of the DES, respectively, was (i) to avoid excessive heating, which could lead to preferential evaporation and thus changes in DES composition, and (ii) to assure that the prepared DES remained in the liquid state under common ambient conditions for which 273 K was considered a reasonable limit. The water content was reduced by vacuum drying at 313 K (Heidolph rotary evaporator), with the water content measured with a Karl Fischer coulometric titrator (Metrohm 831 KF coulometer, ±0.3%) and kept under vacuum. The water content for LEV–[N4444]Cl in a 2:1 mol ratio, for which experimental measurements are reported in this work, was 0.3 wt %.

### 2.2 Apparatus and Procedures

The water absorption from atmospheric humidity was experimentally measured for LEV–[N4444]Cl (2:1). For that purpose, a kinetic experiment was carried out by placing 15 cm^3^ DES samples in a Petri dish (90 mm diameter, 25.5 cm^2^ liquid surface exposed to air) and opened to atmosphere (50  ± 5% relative humidity). Samples (∼0.1 g) were extracted as a function of time, and the water content was measured using Karl Fischer coulometry. The experimental results were fitted to the following kinetic model:
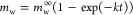
1where *m*_w_ stands for the water content (wt %), *m*_water_^∞^ for the limiting absorption value, *k* for the absorption rate, and *t* for time (in hours).

A collection of relevant thermophysical properties (density, shear viscosity, electrical conductivity, thermal conductivity, and refractive index) were measured for the macroscopic characterization of the DES. Density (ρ, uncertainty ±1 × 10^–4^ g cm^–3^) measurements were carried out using a vibrating tube densimeter (Anton Paar DMA1001), with temperature control (±0.01 K) by an internal Peltier. Shear viscosity (η, uncertainty ±2%) was measured with an electromagnetic piston viscometer (VINCI Tech EV1000).^49^ A circulating external bath (Julabo Presto) was used for temperature control with the cell temperature measured with a platinum resistance thermometer (PRT, ± 0.01 K). The experimental viscosity as a function of temperature was fitted to the Vogel–Fulcher–Taman (VFT) model:
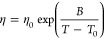
2

The refractive index (sodium D-line, *n*_D_, uncertainty ±1 × 10^–5^) was measured using a Leica AR600 refractometer and an external thermostatic bath (Julabo F32) controlled the cell temperature, which was measured using a PRT. The molar free volume, *f*_m_,^50^ was calculated from experimental *n*_D_. The electrical conductivity (κ, ± 0.5% uncertainty) was measured using a VWR pHenomenal conductivity meter, which was calibrated using certified KCl solutions. A Julabo F32 bath controlled the cell temperature (±0.01 K), which was measured with a PRT. Thermal conductivity (σ, 5% uncertainty) was measured with a Decagon devices KD2 thermal analyzer (KS-1 sensor, 6 cm long, 1.3 mm diameter single needle), considering the minor changes of σ with temperature. This property was measured only at 298.15 K, with the temperature controlled using Julabo F32 and measured using a PRT. The measured thermophysical properties are reported in Table S2 (Supporting Information).

### 2.3 Molecular Modeling

Density functional theory (DFT) studies on [N4444]Cl (2:1) clusters were carried out using TurbomoleX V7.5.1.^51,52^ Clusters were optimized using the BP86^53^ functional combined with def-TZVP basis set and D3^54^ dispersion contribution (semiempirical Grimme’s method). The quantum theory of atoms in molecule (QTAIM)^55^ was used for the topological characterization of the hydrogen bonding in these clusters using the MultiWFN software.^56^ QTAIM considers molecules as composed of individual atoms that are interconnected through the shared electron density. These atoms in molecules are not necessarily the same as the chemical elements but represent regions of a high electron density within the molecule. QTAIM identifies regions in the electron density where electron pairs are localized as bonding regions (associated with covalent bonds) and regions of low electron density as nonbonding regions (associated with nonbonding electron pairs or lone pairs). The theory employs mathematical tools, including the Laplacian of the electron density, to analyze the topology (shape and connectivity) of the electron density and identify critical points, such as bond critical points, where the electron density is at a maximum, and nonbonding critical points, where it is at a minimum. By analyzing the electron density and critical points, QTAIM provides valuable insights into various molecular properties, including bond strengths, bond types, and the presence of weak interactions like hydrogen bonds and van der Waals forces.

The QTAIM analysis of hydrogen bonding is done considering the developed bond critical points (BCPs), along with ring critical points (RCPs) in the interaction region. Additionally, noncovalent interaction analysis (NCI)^57^ was also developed for optimized clusters using MultiWFN. The [N4444]Cl–LEV interaction energies, Δ*E*, were calculated as the difference between the energy of the cluster minus those of the corresponding monomers. The ChelpG,^58^ Lowdin,^59^ and Hirshfeld^60^ were considered to calculate the atomic charges for the optimized clusters. Additionally, the COnductor-like Screening MOdel for Real Solvents (COSMO-RS) was applied to the DFT-optimized structures for the characterization of molecular moieties related to hydrogen bonding. The COSMO-RS approach has proved to be suitable for the characterization of DESs.^61,62^ The COSMOtherm software (version 2021-21.0) was considered for the analysis of hydrogen bonding region distribution in the considered molecules.

Classical MD simulations were carried out using the MDynaMix v.5.2^63^ software for the systems and conditions reported in Table S3 (Supporting Information). Initial simulation boxes, in cubic geometries (Figure S1, Supporting Information), were built using the Packmol^64^ program for densities as in experimental results. The force-field parametrizations for the involved molecules are reported in Table S4 (Supporting Information) and they were obtained from the SwissParam database (Merck Molecular Force Field)^65^ as well as ChelpG charges obtained from DFT simulations of isolated molecules. Periodic boundary conditions in the three space directions were applied for all of the simulations. Simulations were carried out in a three-step procedure: (i) starting from initial boxes, 10 ns NVT simulations at 343 K, (ii) 40 ns NPT simulations, at each selected pressure–temperature condition for equilibration purposes, and (iii) 100 ns NPT production simulations at each selected pressure–temperature condition. Therefore, a total of 150 ns simulations were carried out for each pressure–temperature condition. The experimental viscosity of LEV–[N4444]Cl (2:1) is in the range 636–43 mPa·s for the 293–333 K range, for which MD simulations were carried out. The considered MD total simulation time assured reliable sampling, considering the DES viscosity. The pressure and temperature control along MD simulations were carried out with the Nose–Hoover method,^66,68^ with 30 and 1000 ps as temperature and pressure coupling times, respectively. The Tuckerman–Berne double-time step algorithm (with long- and short-time steps of 1 and 0.1 fs) was used for solving the equations of motion. The electrostatic interactions were handled with the Ewald method (1.5 nm cutoff radius)^67,69^ and the Lennard-Jones potential was applied with a 15 Å cutoff distance, with the Lorentz–Berthelot mixing rules applied for cross terms.

## 3 Results and Discussion

### 3.1 Experimental Properties

LEV-based DES was prepared for different LEV:[N4444]Cl *i*:*j* mole ratios. For all of the studied mole ratios (2.5:1, LEV-rich, to 1:2.5, [N4444]Cl-rich), slightly yellow, moderately viscous fluids were obtained (Figure 1). All of the prepared DES were cooled to 273 K, where they remained liquid for 48 h, thus proving a wide liquid range below the ambient temperature. It should be stated that in spite of the natural origin and nontoxic character of LEV, the second component of the considered DES ([N4444]Cl) has been probed to be toxic (e.g., to human skin cells) both in pure form and upon formation of DES with HBDs such as butanoic acid, hexanoic acid, ethylene glycol, 1-propanol, or urea.^70^ Thus, a certain degree of toxicity may be expected for [N4444]Cl–LEV DES. Therefore, considering that the second component of the DES (LEV) is nontoxic, DES toxicity may be regulated by fine-tuning the HBA:HBD ratio.^71^ The results in Figure 1 indicate liquid DES for a wide range of HBA:HBD molar ratios. A high-LEV-content DES (2:1) was selected for further studies to minimize toxicity and to increase the content of the natural compound in the final DES. Therefore, the LEV:[N4444]Cl 2:1 mol ratio was selected, being rich in LEV and liquid at ambient temperature, and subjected to detailed physicochemical characterization as well as theoretical study in the following sections.

Regarding the possible hydrophobic or hydrophilic nature of [N4444]Cl:LEV (2:1) DES, Florindo et al.^72^ showed leaching of both [N444]Cl and LEV components toward the aqueous phase, thus indicating the hydrophilic nature of the considered DES. Therefore, to quantify this hydrophilicity and considering the water effect on DES properties, the water absorption from atmospheric humidity was measured and fitted to the kinetic model considered in eq 1. The reported results in Figure S2 (Supporting Information) show moderate but non-negligible water capture, with saturation at 6.851 wt %, thus confirming the certain hydrophilic nature of the DES. Likewise, the water absorption kinetics is slow (*k* = 0.146 h^–1^), thus short exposures of the fluid to air should not lead to large changes in water content, therefore maintenance of low water content as the one considered in this work for experimental measurements (0.3 wt %) should be feasible.

**Figure 2:**
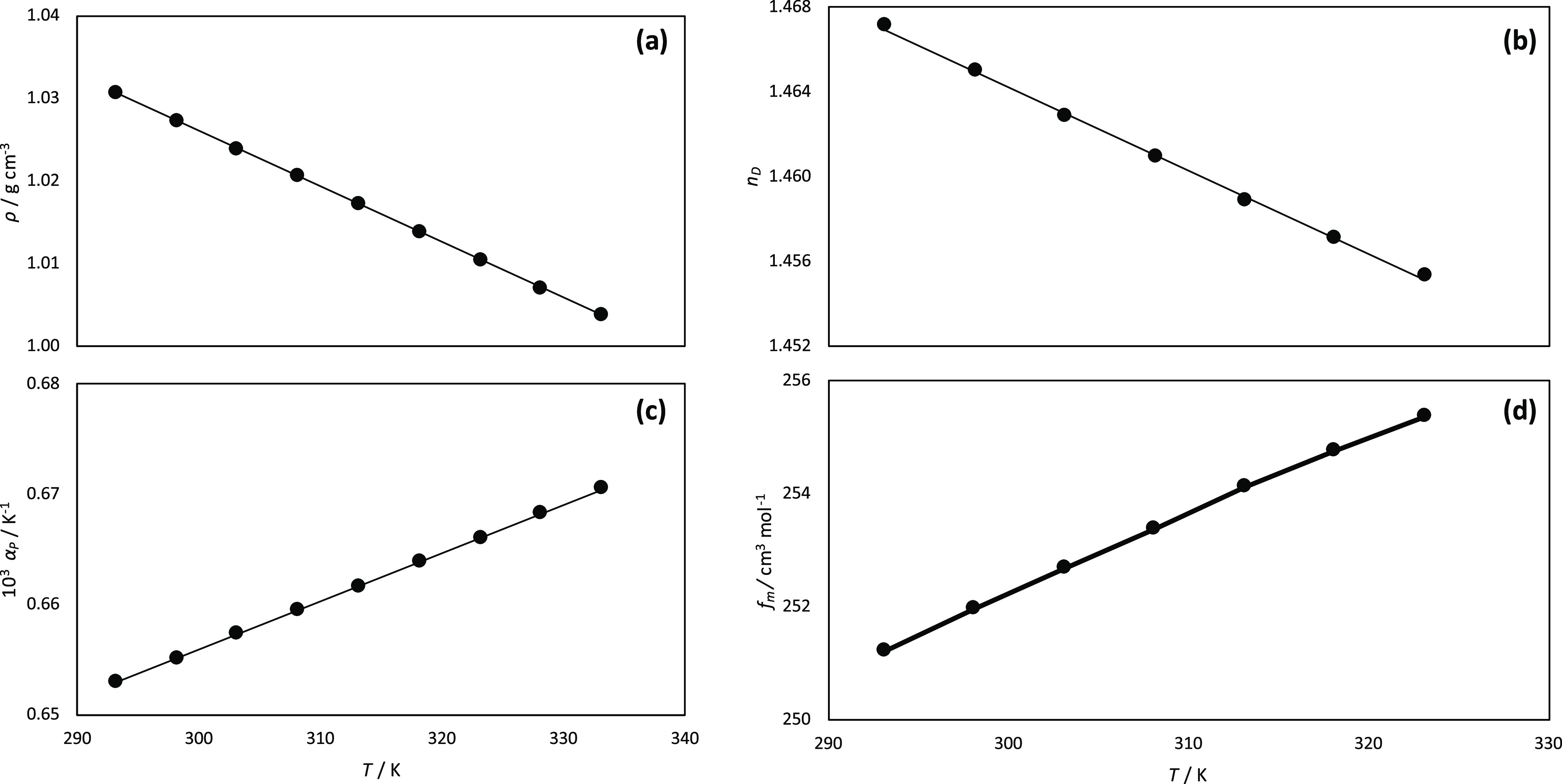
Experimental (a) density, ρ, (b) isobaric thermal expansion coefficient, α_p_, (c) refractive index, *n*_D_, and (d) free volume, *f*_m_, for LEV:[N4444]Cl (2:1) DES as a function of temperature.

Density was measured in the 293.15–333.15 K range (Figure 2a), showing a moderately dense fluid. It should be remarked that hydrophobic DES are less dense than hydrophilic ones showing densities lower than those for water. Considering the certain hydrophilic nature of [N4444]Cl:LEV (2:1), a fluid slightly denser than water is inferred (3.3 and 2.1% denser at 293.15 and 333.15 K, respectively).^73,74^ Li et al. reported the experimental physicochemical properties for [N4444]Cl–LEV DES, including density data; although these authors did not report the exact molar ratio of the studied DES, a certain comparison with the results reported in this work may be carried out. Li et al. values for density were 1.03453 and 1.00600 g cm^–3^ at 293.15 and 333.15 K, respectively, which are 0.38 and 0.22% larger than the values reported in Table S2 (Supporting Information) and thus are in fair agreement with volumetric properties reported in this work.

The refraction index was also measured, showing a linear decrease with increasing temperature (Figure 2b). The evolution of density and refraction index with increasing temperature allowed us to infer the availability of free space in the considered DES. In the case of density, it showed a linear decrease upon heating, which was used for the calculation of an isobaric thermal expansion coefficient, α_p_, eq 3 (Figure 2c).
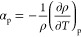
3

Likewise, the experimental *n*_D_ was used to calculate the molar free volume, *f*_m_,^48^ (Figure 2d). The calculated *f*_m_ values are large, which points to poorly efficient molecular packings in the considered DES.
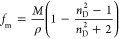
4

The obtained α_p_ values show slightly increasing values with temperature, following a linear trend. A comparison is reported in Table 1 for the volumetric properties of different LEV-based DES, including the previous works by Ullah et al.,^75^ Gajardo-Parra et al.,^76^ Gutiérrez et al.,^77^ and Sánchez et al.^78^ The reported results indicate densities larger than those of water for the considered DES, with the exception of some involving hydrophobic HBAs (e.g., menthol, LEV), with density values for [N4444]Cl–LEV being remarkably lower than most of the considered LEV-based DES. Regarding α_p_ values for [N4444]Cl–LEV, they are in the range of all the considered LEV-based DES being lower (i.e., less compressible fluid) than those involving hydrophobic HBAs (e.g., menthol–thymol), and thus this low compressibility being a characteristic of LEV-based DES involving hydrophilic HBAs. The free volume values are also in the same range for all of the considered LEV-based DES involving hydrophilic HBAs.

**Table 1:** Comparison between Experimental Properties Obtained in This Work at 293.15 K and Those from Relevant Selected Literature

	ρ (g cm^–3^)	10^3^α_P_ (K^–1^)	*f*_m_ (cm^3^ mol^–1^)	η (mPa·s)	ref
[N4444]Cl–LEV (1:2)	1.0367	0.653	250.5	636.0	this work
[choline]Cl–LEV (1:2)	1.1419	0.583	236.41^a^	362.08	(75,76)
menthol–LEV (1:1)	0.98624	0.795	102.3	36.8	(77)
thymol–LEV (1:1)	1.04183	0.777	91.0	41.9	(77)
l-proline–LEV (1:2)	1.20100	0.577	207.64	955.0^b^	(78)
betaine–LEV (1:2)	1.16190	0.602	217.38	1342^c^	(78)

a Data at 298.15 K.

b Data at 303.15 K.

c Data at 298.15 K.

**Figure 3:**
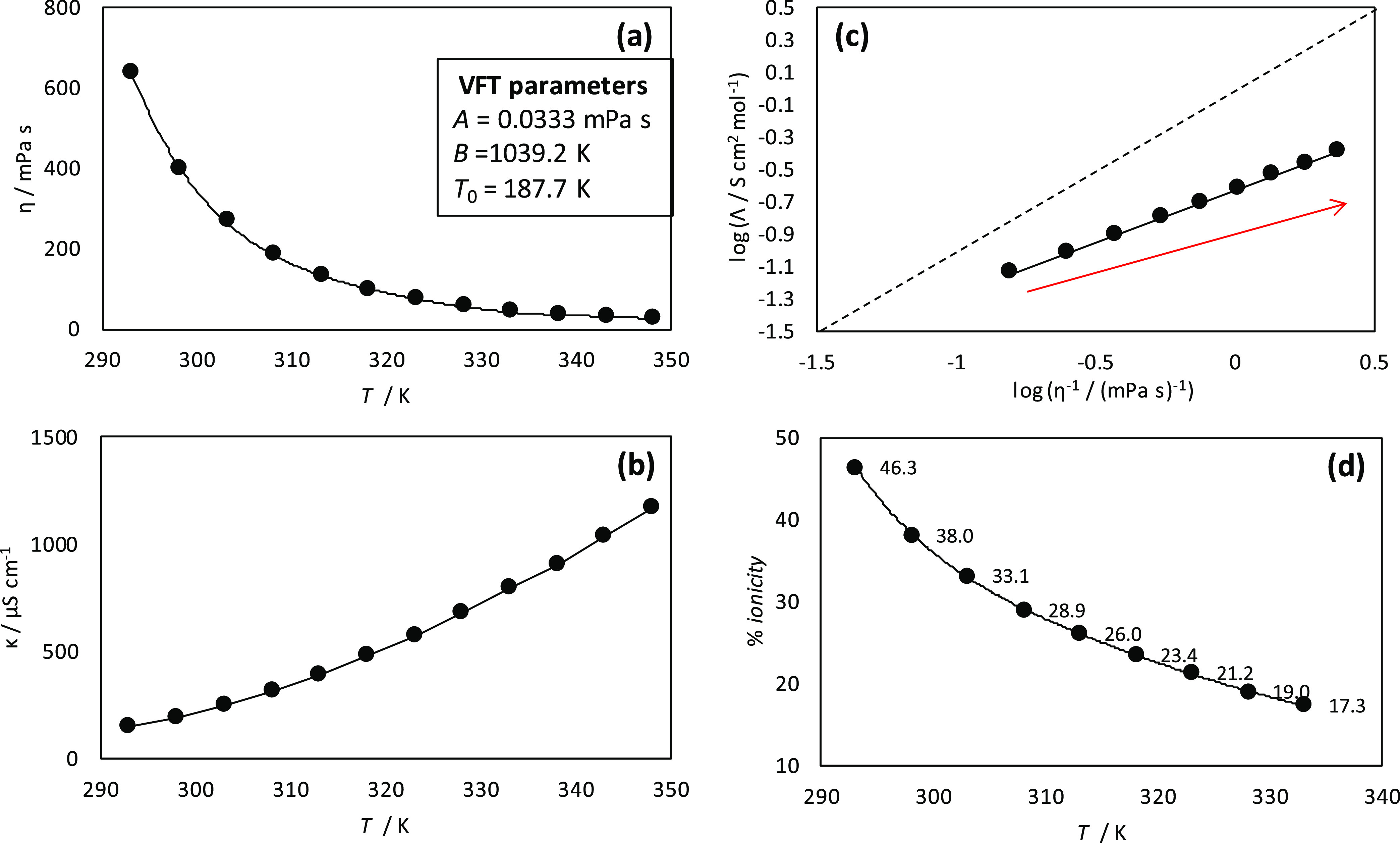
Experimental (a) dynamic viscosity, η, and (b) electrical conductivity, κ, for LEV:[N4444]Cl(2:1) DES as a function of temperature. Panel (c) reports Walden plot showing the log–log plot of molar conductivity, Λ, vs fluidity, η^–1^ (where η stands for dynamic viscosity). Panel (d) shows the calculated percentage of ionicity as inferred from eq 6. VFT fit parameters of experimental viscosity are reported in panel (a). Dashed line in panel (c) shows the reference theoretical behavior of 0.01 M KCl solution. A red arrow in panel (c) shows increasing temperature.

Dynamic viscosity reported in Figure 3a shows that the considered DES is a viscous fluid, which falls within the range of typical type III DES considering the size of the considered cation and the hydrogen bonding ability of the HBD (LEV). The viscosity values reported by Li et al.^74^ are remarkably lower than those reported in this work, which are 121.68 and 17.47 mPa s at 293.15 and 333.15 K, respectively, compared with 636 and 42.7 mPa s, respectively (Table S2, Supporting Information). The reasons for this discrepancy may be the molar ratio of the DES (not stated in Li et al.^74^ work) or the method of preparation or viscosity measurements. Likewise, Table 1 shows a comparison of several LEV-based DES, and these results show that LEV-based DES considering hydrophilic HBAs (e.g., [N444]Cl) are remarkably more viscous than those involving hydrophobic HBAs (e.g., menthol or thymol). Nevertheless, the decrease in [N4444]Cl–LEV viscosity upon heating would lead to reasonable viscosity at temperatures slightly above ambient conditions, which is of relevance for practical purposes. Likewise, the temperature effect on viscosity follows a non-Arrhenius behavior, which was fitted to the VFT model. The *T*_0_ VFT parameter is related to the glass transition temperature^79^ and a low value confirms the wide liquid range of this DES. Angell’s fragility parameter, *D*, is defined by VFT coefficients according to eq 2. A *D* value of 5.54 is obtained for [N4444]Cl:LEV (2:1) DES, which indicates a fragile fluid.
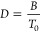
5

Electrical conductivity was also measured (Figure 3b), showing a poorly conductive fluid at temperatures close to ambient temperature because of the viscous behavior but with a large increase upon heating, considering the increasing mobility of charge carriers. The electrical conductivity values reported in this work are lower than those reported by Li et al.,^74^ which agrees with the larger viscosity data. The Walden plot was considered (Figure 3c) for the common analysis of viscosity and electrical conductivity, which was also considered for calculating the ionicity as a function of temperature^80^ (Figure 3d). The percentage of ionicity (% ionicity) was calculated considering eq 6:
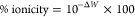
6where Δ*W* is defined as the vertical distance in the Walden plot from each point to the 0.01 M KCl reference line. The Walden plot shows a linear behavior with increasing temperature and indicates the poor ionic nature of the considered DES. The DES ionicity follows a nonlinear trend with increasing temperature and decreases upon heating, which has been previously reported for other type III DES.^80^ As temperature increases, two main factors should be considered to explain the evolution of ionicity: (i) density decreases upon heating and so does molar concentration, which should decrease the electrostatic interaction between ions, and thus increasing ionicity; nevertheless, we will show in the following theoretical section that the weakening of the cation–anion Coulombic interaction is almost negligible in the considered temperature range; (ii) increase of diffusion rates upon heating favors intermolecular contacts and thus interionic association, thus decreasing ionicity. These two opposite factors should control ionicity evolution with temperature. Thus, considering that the strength of cation–anion interactions is only slightly disrupted upon heating, it seems that the increase in molecular motion leads to the decrease in ionicity reported in Figure 3d. Therefore, the large HBD content in the considered DES, as well as the developed HBA:HBD hydrogen bonds should be the reason for the poor ionicity of the considered fluid.

### 3.2 Molecular Simulations

**Figure 4:**
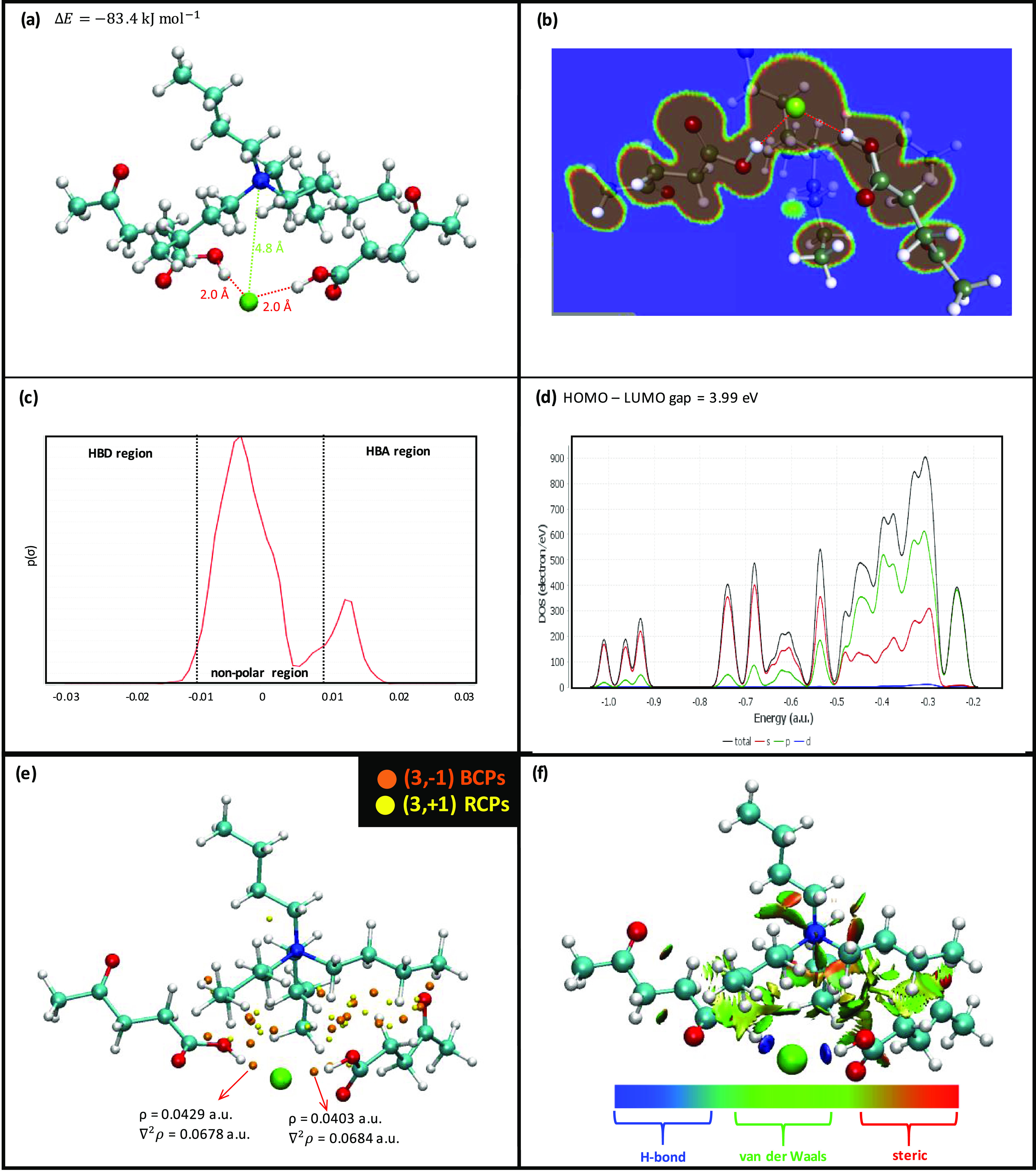
DFT results for LEV:[N4444]Cl(2:1) DES cluster at BP86-d3/def-TZVP theoretical level. (a) Optimized structure of the cluster, reporting interaction energy, Δ*E*, OH(LEV)–Cl hydrogen bonds (red dashed lines), and Cl–N([N4444]) (green dashed line). (b) Electron localization function in the plane containing OH(LEV)–Cl hydrogen bonds. (c) σ-profiles calculated from the COSMO-RS method. (d) Density of states (DOS) indicating different contributions. (e) AIM. (f) NCI analysis.

The results in the previous section showed how the studied DES is a moderately dense and viscous fluid, with available free space and poor ionicity, thus pointing to strong HBA:HBD hydrogen bonding. The nanoscopic nature and origin of this macroscopic behavior were analyzed using molecular simulations. In the first stage, DFT calculations of HBA:HBD clusters were carried out for a detailed characterization of hydrogen bonding using minimal cluster models (Figure 4a). The optimized structure of the cluster probed HBA:HBD interaction via Cl–LEV hydrogen bonding, with both LEV molecules interacting with the anion in equivalent positions. The strength of the interaction quantified through the large Δ*E* should be at the root of the properties such as high viscosity or poor ionicity reported in the experiments. Likewise, the structure of the cluster shows a bulky structure with a cation alkyl chain on top of the plane on which the anion–LEV hydrogen bonding is produced, which should lead to a large free volume (Figure 2d) in the liquid when this type of cluster is produced. The analysis of hydrogen bonding regions was also done using the electron localization function (ELF) (Figure 4b), which shows a large accumulation in the region around the developed hydrogen bonds. The core–valence bifurcation index (CVB)^81^ was calculated from ELFs for the corresponding hydrogen bonding atoms, being −0.1480 and −0.1310 for the two developed Cl–LEV hydrogen bonds. Large negative CVB values are obtained for the developed hydrogen bonds, which indicate strong interactions.^82^ Fan et al.^83^ proved a direct relation between CVB and viscosity, with larger (in absolute value) CVB corresponding to more viscous DES. Thus, the CVB values obtained for [N4444]Cl:LEV would justify the viscous behavior reported in Figure 3a, although steric effects arising from the hindered ionic diffusion because of the bulky clusters should also be considered. The σ-profiles obtained for the optimized cluster and reported in Figure 4c show the prevailing nonpolar character of the studied cluster because of the presence of the [N4444] cation, with the presence of HBA regions (Cl anion) and minor HBD regions because of the very localized nature of hydrogen bonds around the LEV-hydroxyl groups. The calculated density of states (DOS) is reported in Figure 4d, showing a moderate highest occupied molecular orbital (HOMO)–least unoccupied molecular orbital (LUMO) gap (3.99 eV), which is in the range of previously reported DES,^84^ thus indicating stable clusters via hydrogen bonding in the liquid DES structure. Further analysis of the hydrogen bonding was carried out using the QTAIM approach (Figure 4e). The QTAIM analysis showed the appearance of BCPs and RCPs in the HBA:HBD interacting regions. The BCPs corresponding to the anion–LEV hydrogen bonding (red arrows in Figure 4e) indicate very strong interactions. According to the Popelier criteria,^85^ hydrogen bonds correspond to ρ (electron density) and ∇^2^ρ for the corresponding BCPs in the 0.002–0.04 and 0.020–0.139 au ranges, respectively, with the larger values corresponding to stronger hydrogen bonds. Therefore, the Cl–LEV hydrogen bonds are strong interactions, which agrees with the large Δ*E* and CVB values. Moreover, the HBA:HBD interaction is characterized not only by the development of hydrogen bonds through the anion, the large number of developed BCPs as well as the reported RCPs in the region between [N4444] alkyl chains and the LEV molecules indicate that remarkable van der Waals interactions are also developed, which would contribute to the effective HBA:HBD interaction. These effects are confirmed through the NCI analysis reported in Figure 4f, which apart from the localized spots in the region between the hydrogen donor and acceptors shows large regions corresponding to van der Waals interactions. Once strong van der Waals and hydrogen bonding interactions are confirmed, the ionic nature of the considered HBD should require the analysis of the charges, which would lead to neat electrostatic interactions. Total charges in the [N4444] cation, Cl anion, and LEV were calculated using three different methods and reported in Table 2. In spite of the differences between the charges obtained with the different methods, all of the results indicate cation/anion charges very different from those from +1/–1. Likewise, LEV molecules are also charged. Therefore, the formation of the HBA:HBD cluster leads to charge transfer, with the low negative charge on the Cl anion indicating transference from this anion toward the LEV molecules as well as having an effect on the cation’s total charge.

**Table 2:** Molecular Charges, *q*, for the LEV:[N4444]Cl(2:1)-optimized cluster (Figure 7) Using Different Charge Definition Models^a^

molecule	*q* (Löwdin)	*q* (Hirshfeld)	*q* (ChelpG)
[N4444]^+^	+0.8933	+0.7413	+0.8996
Cl^–^	–0.5865	–0.4907	–0.7681
LEV1	–0.1559	–0.1132	–0.0270
LEV2	–0.1510	–0.1374	–0.1045

a LEV1 and LEV2 stand for the two different LEV molecules forming the cluster.

**Figure 5:**
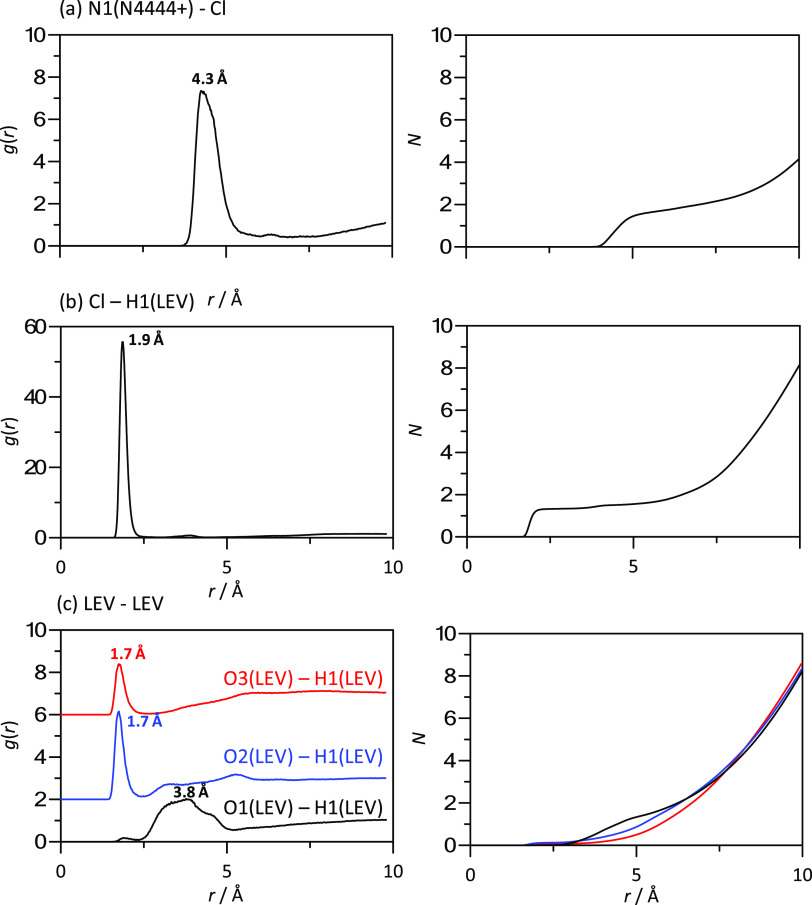
Site–site radial distribution functions, *g*(*r*), and the corresponding running integrals, *N*, for LEV:[N4444]Cl(2:1) DES from MD simulations at 293 K and 1 bar. Atom labeling is the same as in Figure 1.

**Figure 6:**
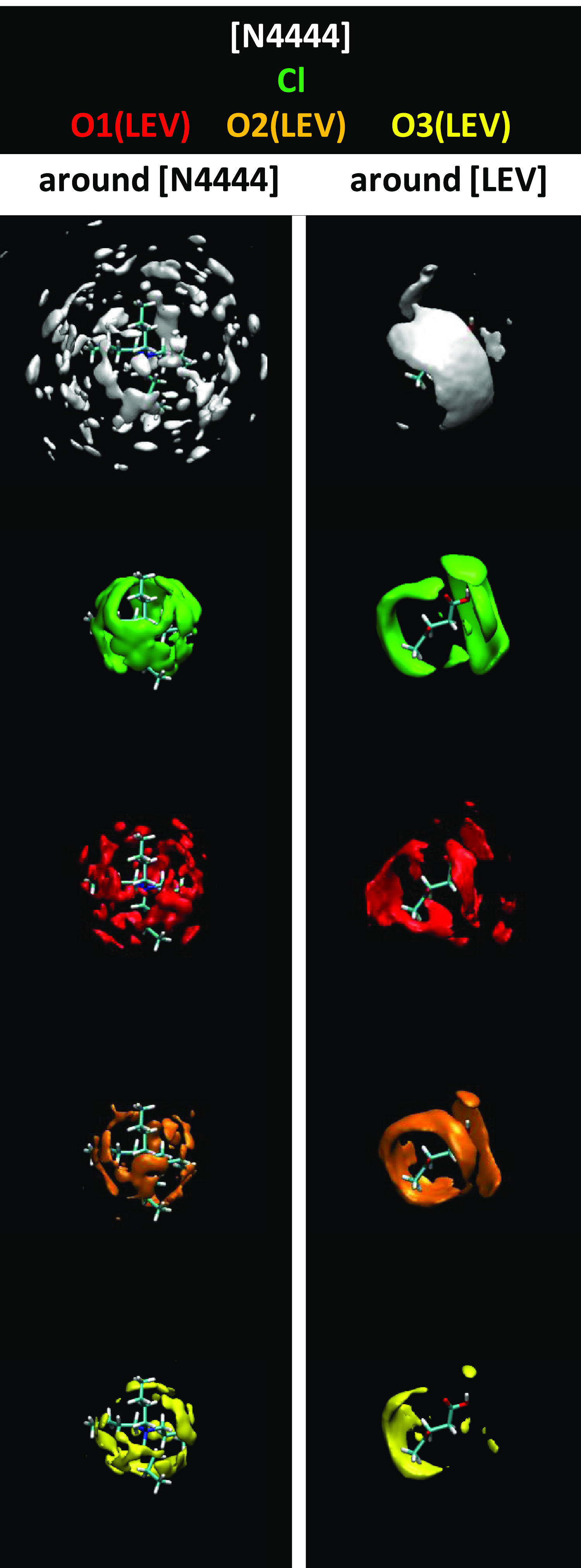
Spatial distribution functions around [N4444] and LEV molecules for LEV:[N4444]Cl(2:1) DES from MD simulations at 293 K and 1 bar. Atom labeling is the same as in Figure 1.

**Figure 7:**
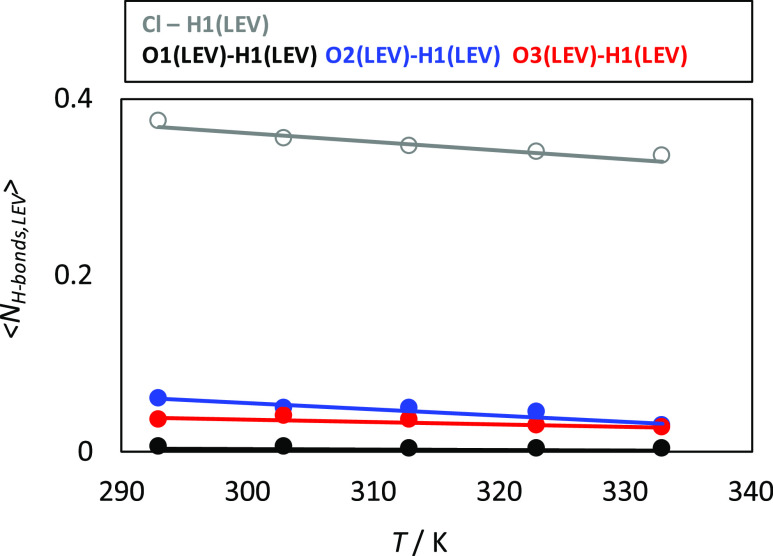
Average number of hydrogen bonds per LEV molecule for the reported atomic pairs in LEV:[N4444]Cl(2:1) DES from MD simulations at 1 bar as a function of temperature. Atom labeling is the same as in Figure 1. Hydrogen bonding criteria: 3.5 Å and 60° as donor–acceptor distance and angle.

The DFT results provided information on the hydrogen bonding properties, but further information on bulk liquid phase properties at the nanoscopic level was obtained from MD simulations. The force-field parametrization considered is analyzed by comparing the predicted density data with experimental values, with results in Figure S3 (Supporting Information) indicating fair agreement. Although MD underestimates density, percentage deviations with experimental results are lower than ∼0.5% in the studied range 293–333 K, which allows us to reliably predict the capabilities of the studied systems. The initial analysis of nanoscopic structuring was carried out using suitable radial distribution functions (RDFs) as well as the corresponding running integrals, which allows us to determine the composition of solvation shells around relevant atomic sites (Figure 5). The reported results confirm the cation–anion interaction (Figure 5a) characterized by strong Coulombic interactions, accompanied by Cl–LEV hydrogen bonding through the LEV-hydroxyl site, leading to interaction distance equivalent to those from DFT studies (2 Å) (Figures 4a and 5b). Regarding the LEV–LEV self-association (Figure 5c), as the LEV-hydroxyl site (O1 oxygen atom, Figure 1) is occupied by the interaction with Cl anion, the interaction between neighbor HBA:HBD clusters are developed through the CO sites, which are hydrogen bonded with OH sites and thus the bulk liquid structure will be characterized by clusters as reported in DFT results (Figure 4a) connected through LEV–LEV CO···OH hydrogen bonds. Further analysis is obtained from the spatial distribution functions (SDFs, Figure 6). The distribution around the [N4444] cation is characterized by almost spherical symmetry for all of the possible molecules, whereas a highly localized distribution is inferred for LEV, with Cl around the hydroxyl sites and neighbor molecules in areas not occupied by the anion close to the LEV CO groups, thus allowing simultaneous LEV–Cl and LEV–LEV hydrogen bonding. As the main feature for [N4444]Cl:LEV structuring rises from the anion–LEV hydrogen bonding, the true nature of this interaction was confirmed by calculating the combined distribution function (CDF) involving the distance and angle for the corresponding donor and acceptor sites. CDF results (Figure S4, Supporting Information) confirm this interaction as a true hydrogen bond with an almost donor–acceptor linear orientation in agreement with DFT results for isolated clusters, thus the presence of neighbor competing hydrogen bonding sites does not disrupt the structure of the minimal clusters as the availability of CO sites in LEV allows an effective interconnection of clusters without disrupting the anion–HBD interactions. The average number of hydrogen bonds per (LEV) molecule was calculated for different pairs as a function of temperature (Figure 7). The prevailing Cl–anion interaction is confirmed as well as the LEV–LEV hydrogen bonding through the CO sites. Likewise, hydrogen bonding is not largely disrupted upon heating in the studied temperature range (up to 333 K); thus, the fluid structure is mostly maintained.

**Figure 8:**
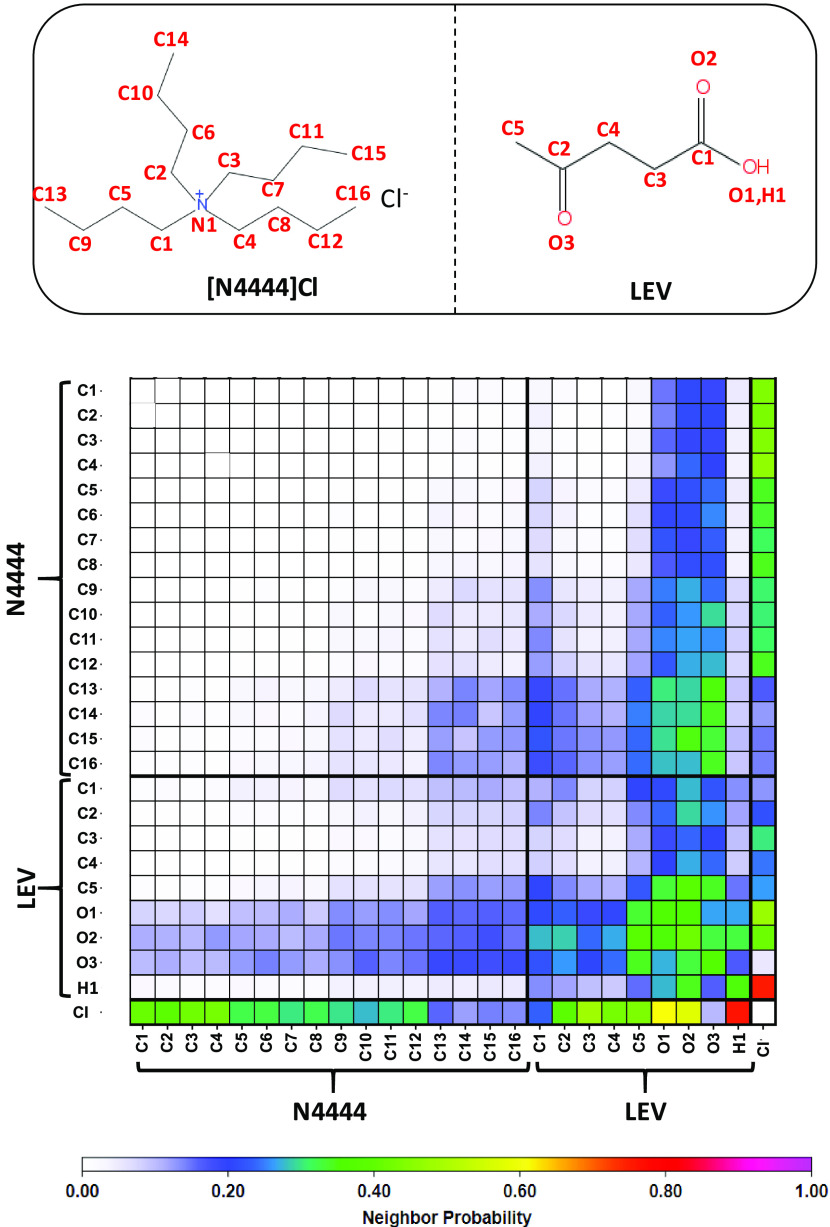
Neighborhood probability matrix from the Voronoi analysis in LEV:[N4444]Cl(2:1) DES from MD simulations at 298 K and 1 bar.

Beyond the analysis of hydrogen bonding, additional information on the fluid’s structuring was inferred from the neighborhood analysis obtained from the Voronoi tessellation.^86^ Therefore, the results in Figure 8 indicate the neighborhood probabilities for relevant atomic sites. The neighborhood analysis indicates the anion–LEV hydrogen bonding as well as LEV–LEV interaction. Likewise, these results confirm the development of LEV–[N4444] and LEV–LEV intermolecular interactions through alkylic chains, i.e. van der Waals contacts, which contributes to the stabilization of the HBA:HBD interactions beyond the neat hydrogen bonds are also inferred from NCI results (Figure 4c).

**Figure 9:**
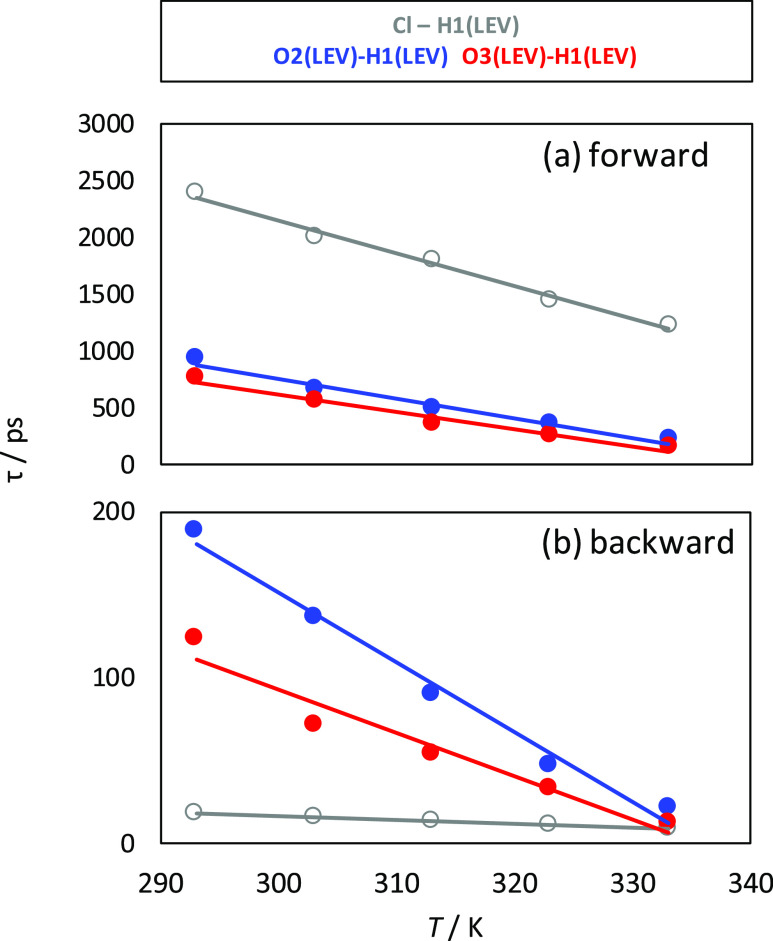
Dynamics of hydrogen bonds for the reported atomic pairs in LEV:[N4444]Cl(2:1) DES from MD simulations at 1 bar as a function of temperature. Atom labeling is the same as in Figure 1. Results in panel (a) show time, τ, for a forward process (i.e., hydrogen bond lifetime) and in panel (b) for a backward process (i.e., formation of hydrogen bonding after breaking).

The dynamic of the hydrogen bond formation–destruction was analyzed through the reactive flux analysis,^86^ which allowed us to calculate the hydrogen bonding lifetime (so-called forward process) as well as time for interaction reforming after breaking (backward process) (Figure 9). Results in Figure 9a indicate large lifetimes for all of the developed hydrogen bonds, although times for Cl–LEV interactions are almost 3 times larger than that for LEV–LEV ones. Lifetimes decrease upon heating, although anion–LEV ones retain hydrogen bonding even at higher temperatures. Another remarkable feature of the developed hydrogen bonds is the short time required for hydrogen bonding to be reformed after breaking (Figure 9b). In the case of anion–LEV interactions, less than 20 ps is required to rebuild a hydrogen bond, whereas a slightly larger time is required to rebuild LEV–LEV interactions but reforming is also fast.

**Figure 10:**
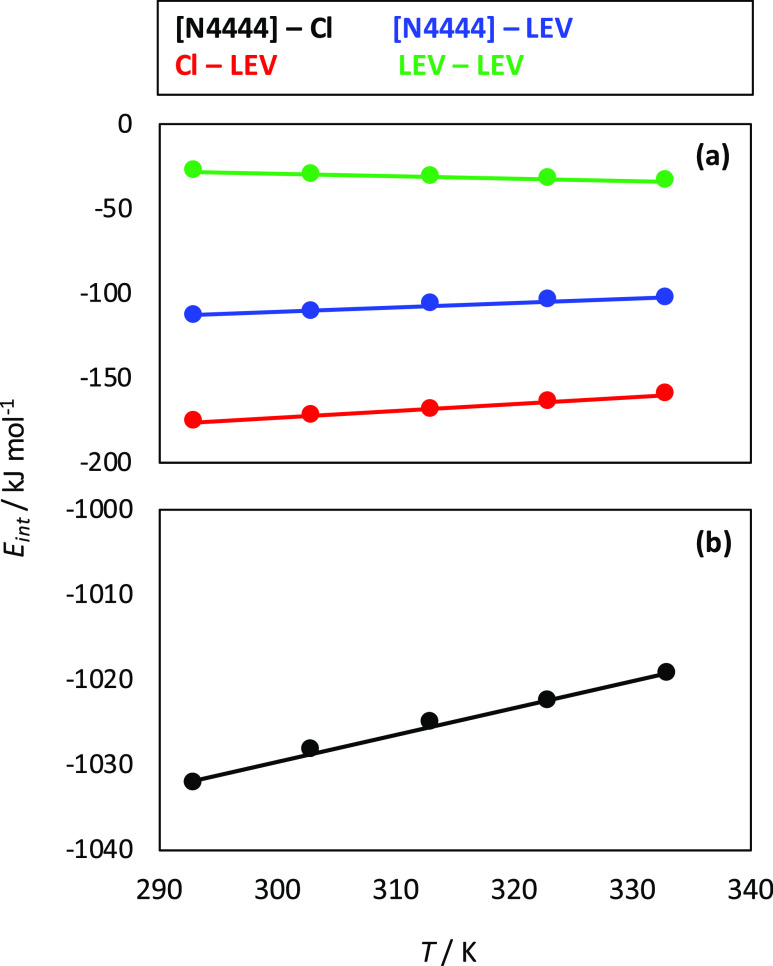
Intermolecular interaction energy, *E*_int_, for the reported molecular pairs for LEV:[N4444]Cl(2:1) DES from MD simulations at 1 bar as a function of temperature.

**Figure 11:**
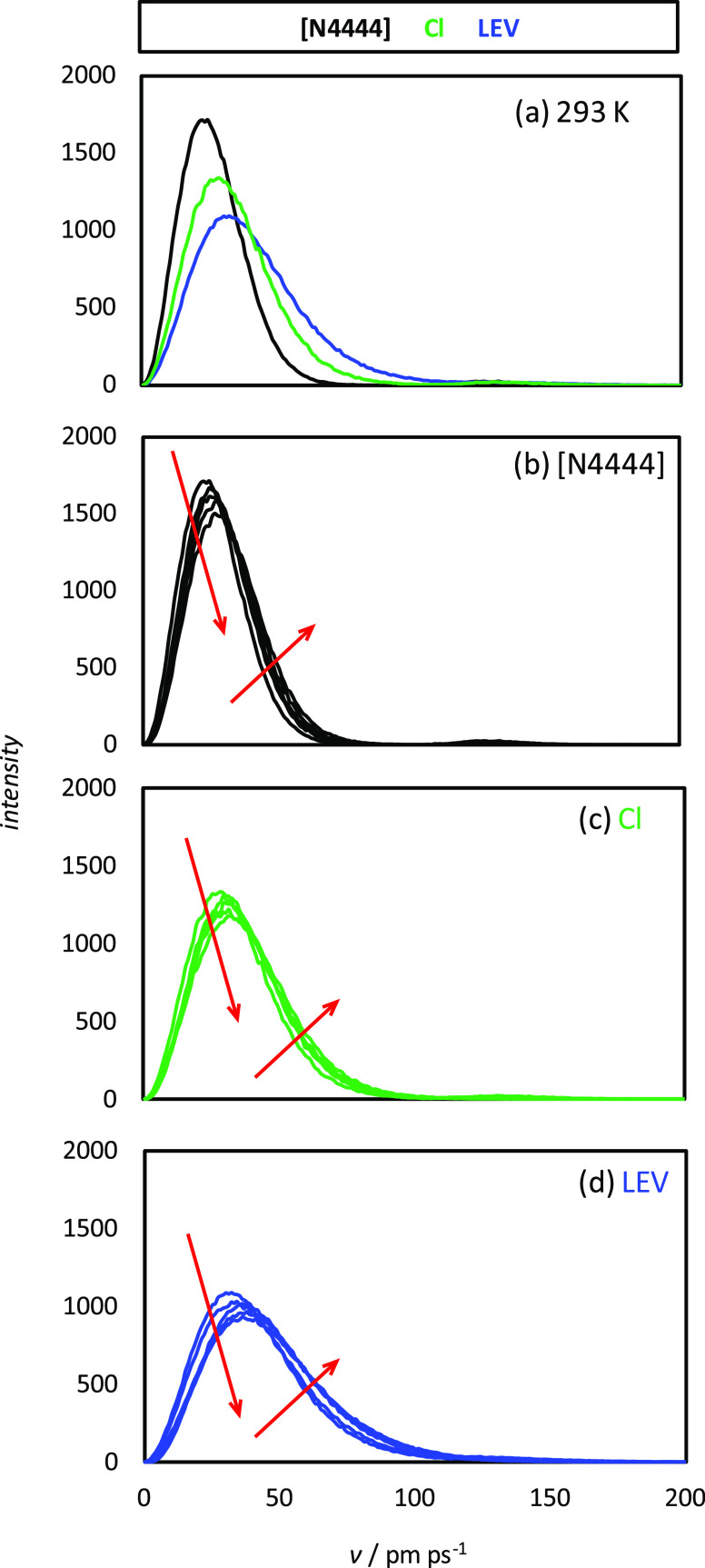
Velocity, *v*, distribution functions for the reported molecules in LEV:[N4444]Cl(2:1) DES from MD simulations at 1 bar. Results in panel (a) are for 293 K, whereas results in panels (b) to (d) show the effect of temperature in the 293–333 K range (10 K steps). Arrows indicate increasing temperature.

**Figure 12:**
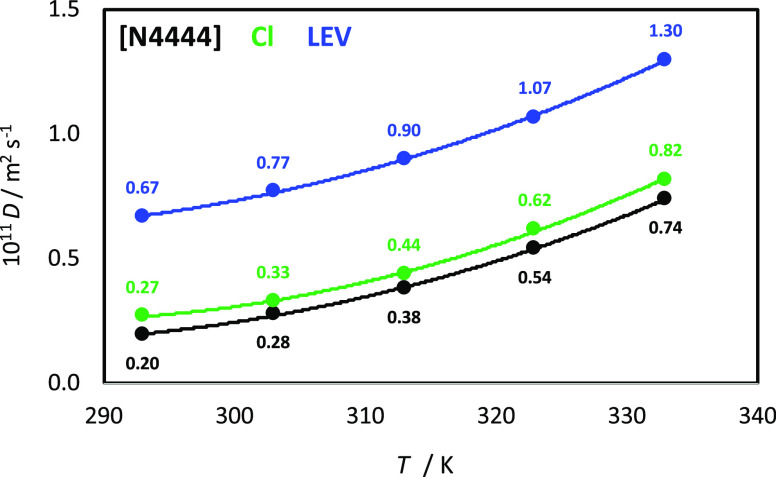
Center-of-mass self-diffusion coefficients, *D*, for the reported molecules in LEV:[N4444]Cl(2:1) DES from MD simulations at 1 bar as a function of temperature.

The strength of the interactions is quantified through the intermolecular interaction energy, *E*_int_ (Figure 10). The reported *E*_int_ for [N4444]–Cl shows large values with almost negligible weakening upon heating because of its Coulombic nature. This small decrease in cation–anion *E*_int_ (1.3% in the considered temperature range) is in agreement with the decrease in ionicity reported in Figure 3d, showing that factors of correlation/anticorrelation motions, size, and dilution effects as well as limitations of the Walden rule are at the root of the reported changes in ionicity. The strength of interactions follows the order Cl–LEV > [N4444]–LEV > LEV–LEV with a minor temperature effect. The dynamics of molecular movement was analyzed with speed following the order [N4444] < Cl < LEV, which is related to the size of the molecules as well to the developed intermolecular interactions (Figure 11). The temperature effect of molecular velocities shows how heating increases molecular velocities, although small changes are inferred, which is related to the minor weakening of intermolecular interactions, Figure 10. The molecular diffusion is also quantified through the calculated self-diffusion coefficients, *D*, obtained from mean square displacements and Einstein’s equation (Figure 12). The *D* values are very similar for the cation and anion because of ion pairing (Figure 10) and slower than that for LEV, with diffusion rates increasing upon heating in a nonlinear way, in agreement with experimental viscosities (Figure 3a).

**Figure 13:**
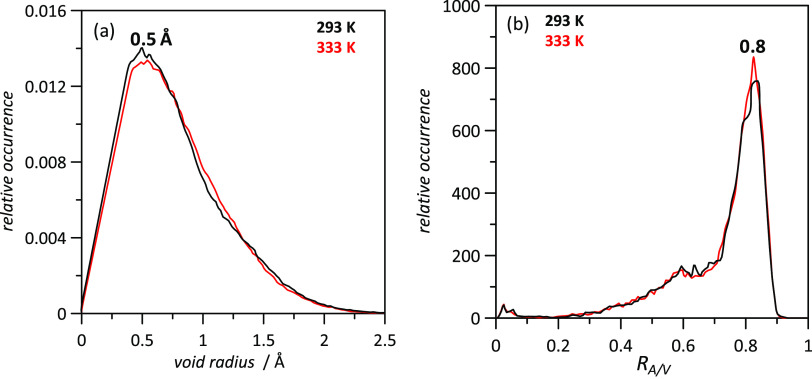
Analysis of void distribution in LEV:[N4444]Cl(2:1) DES from MD simulations at 1 bar as a function of temperature. Panel (a) shows the distribution of cavities as a function of cavity radius. Panel (b) shows the distribution of the isoperimetrical ratio for cavities, *R*_A/V_.

The experimental results indicated the presence of relevant free volume in the studied DES (Figure 2d). This effect was also analyzed considering void distribution inferred from the Voronoi analysis.^86,87^ The reported void distribution shows a Gaussian shape with maxima at 0.5 Å, which was almost unchanged with an increase in temperature (Figure 13a). The geometrical distribution of voids was analyzed using the so-called isoperimetrical ratio for cavities, which quantifies the connection of available cavities with values in the 0–1 range, with values close to 0 indicating connected voids and those close to 1 indicating isolated voids. Results in Figure 13b indicate maxima for the isoperimetrical ratio at 0.79, indicating poorly connected cavities; thus, the available free volume would be formed by isolated spherical cavities, which are distributed around the alkyl chains of the cation. The molecular distribution was also analyzed considering domain analysis^88^ with the Voronoi-based method (Table 3). The domain count number for the cation and LEV is close to unity, which indicates an extended network for these species, as indicated by the volume and surface of their corresponding domains, whereas anions are clearly isolated as they are placed in isolated units inside the HBA:HBD clusters. Likewise, the calculated isoperimetric quotient, which indicates the sphericity of the molecular domains (the lower the value, the more spherical the domain), indicates that cation and LEV domains are clearly nonspherical. Therefore, [N4444]Cl:LEV DES is characterized by large domains with a highly interconnected structure, because of the hydrogen bonding and the presence of alkyl chains but with available free space. Nevertheless, the free space is poorly interconnected but it could be rearranged in the presence of suitable solutes.

**Table 3:** Domain Analysis for LEV:[N4444]Cl(2:1) DES at 298 K^a^

unit	domain_count	D-vol (Å^3^)	D-surf (Å^2^)	*Q*^peri^
[N4444]^+^	1.0	128,442	63,203	0.08
Cl	250.0	35	58	0.85
LEV	1.3	83,015	52,184	0.17

a The domain count is reported with domain volume (D-vol) and surface (D-surf) as well as the isoperimetric quotient (*Q*^per^).

## 4 Conclusions

A combined experimental and theoretical approach analyzed the properties of tetrabutylammonium chloride: levulinic acid natural deep eutectic solvent. The reported results showed a poorly ionic, moderately viscous, and dense fluid with properties primarily determined by the developed efficient hydrogen bonds. The fluid structure has its roots in anion–levulinic acid hydrogen bonding, which leads to minimal clusters interconnected through acid–acid hydrogen bonds. The formed strong hydrogen bonds have long lifetimes and are rapidly reformed after breaking. Likewise, the liquid structure is formed by interconnected nonspherical domains formed by cations and levulinic acid molecules with isolated voids leading to remarkable available free space, which should be relevant for gas absorption and solvation purposes.

## Supporting Information Available

Chemicals considered in this work (Table S1); experimental thermophysical properties (Table S2); systems for MD simulations (Table S3); force-field parametrizations for MD simulations (Table S4); simulation box for MD simulations (Figure S1); water absorption kinetics (Figure S2); comparison of experimental and predicted by MD density (Figure S3); and combined distribution function from MD simulations (PDF)
