# Supplementary material for: Nature of a Tetrabutylammonium Chloride–Levulinic Acid Deep Eutectic Solvent
Source: Ind Eng Chem Res. 2023 Nov 15;62(47):20412–26. doi: 10.1021/acs.iecr.3c02102 (PMC10690803; doi:10.1021/acs.iecr.3c02102)
Supplement: Supplementary file 1 — ie3c02102_si_001.pdf [file ie3c02102_si_001.pdf]

Supporting Information

On the nature of tetrabutylammonium chloride-levulinic acid deep eutectic solvent

Alberto Gutiérrez,[a] Sara Rozas Azcona,[a] Lorena Zamora Pastor,[a] Cristina Benito,[a] Mert Atilhan,[b*] Santiago Aparicio[a*]

[a] *Department of Chemistry, University of Burgos, 09001 Burgos, Spain*

[b] *Department of Chemical and Paper Engineering, Western Michigan University, Kalamazoo MI 49008-5462, USA*

*Corresponding authors: mert.atilhan@wmich.edu (M.A.) and sapar@ubu.es (S.A.)

Table S1. Specifications of the considered chemicals.

chemical name	molar mass (g/mol)	purity (mass%)	source	CAS number
LEV	116.12	99.6 %	Sigma-Aldrich	123-76-2
[N4444]Cl	277.92	99.5 %	Sigma-Aldrich	1112-67-0

Table S2. Thermophysical properties of LEV : [N4444]Cl DES 2 : 1 as a function of temperature at atmospheric pressure. Density, *ρ*, isobaric thermal expansion coefficient, α_p, refractive index, n_D, dynamic viscosity, *η*, and electrical conductivity, *κ*.

T / K	ρ / g cm^{-3}	$10^3\alpha_p$ / K	n_D	η / mPa s	κ / μS cm^{-1}
293.15	1.03067	0.653	1.46713	636.0	147.1
298.15	1.02733	0.655	1.46498	397.5	192.5
303.15	1.02391	0.657	1.46289	267.9	248.0
308.15	1.02054	0.659	1.46094	183.4	315.0
313.15	1.01715	0.662	1.45885	131.9	393.0
318.15	1.01382	0.664	1.45707	97.1	479.0
323.15	1.01047	0.666	1.45535	73.0	576.0
328.15	1.00708	0.668	—	55.3	677.0
333.15	1.00378	0.670	—	42.7	796.0
338.15	—	—	—	34.2	906.0
343.15	—	—	—	28.0	1035.0
348.15	—	—	—	24.5	1175.0

Table S3. Systems considered for molecular dynamics simulations of LEV : [N4444]Cl DES 2 : 1 as a function of temperature at atmospheric pressure. *N* stands for the number of molecules of each type, N_{atoms} for the total number of atoms used in each system, and *L* for the initial dimensions of the cubic simulation boxes in the studied temperature range.

Compound A	Compound B	N(A)	N(B)	N_{atoms}	p / bar	T / K	L / Å
LEV	[N4444]Cl	500	250	21500	1	293, 303, 313, 323,333, 343	59 × 59 × 59

Table S4. Forcefield parameterization for compounds studied in this work.

The general form of the applied force field is:

$$E = \sum_{bonds} k_r (r - r_{eq})^2 + \sum_{angles} k_\theta (\theta - \theta_{eq})^2 + E_{tor}$$
$$+ \sum_i \sum_j \left\{ 4\varepsilon_{ij} \left[\left(\frac{\sigma_{ij}}{r_{ij}} \right)^{12} - \left(\frac{\sigma_{ij}}{r_{ij}} \right)^{6} \right] + \frac{q_i q_j e^2}{4\pi\varepsilon_0 r_{ij}} \right\}$$

Dihedrals (E_{tor}) were described according to:

$$E_{tor} = \sum_{torsions} k_\phi (1 + \cos(m\phi - \delta))$$

Improper dihedrals were described according to:

$$E_{improper} = k_\phi(\phi - \phi_0)^2$$

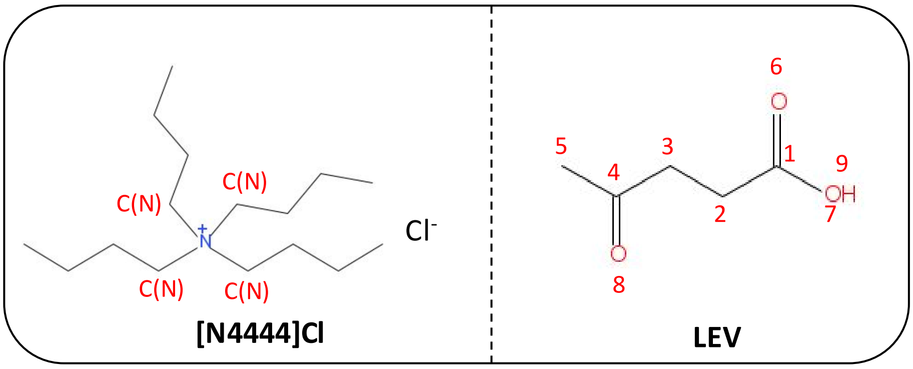

atom	q	σ / Å	ε / kJ mol^{-1}
		[N4444]+	
N	-1.012	3.2963	0.8368
C(N)	0.503	3.8754	0.2301
remaining C	0.000	3.8454	0.2301
remaining H	0.000	2.3520	0.0921
		Cl^-	
Cl	-1.000	3.5000	0.9276
		LEV	
1	0.7140	3.5636	0.4602
2	-0.1591	3.8754	0.2301
3	-0.1246	3.8754	0.2301
4	0.7219	3.5636	0.4602
5	-0.6191	3.8454	0.2301
6	-0.5651	3.0291	0.5021
7	-0.6224	3.1538	0.6364
8	-0.5489	3.0291	0.5021
9	0.4314	0.4000	0.1925
remaining H	0.1103	2.3520	0.0921

# bonds

bond	r_{eq} /Å	k_r / kJ mol^{-1} Å^{-2}
	$[N4444]^+$	
N – C(N)	1.48	1157.5
C(N) – C	1.51	1282.1
C – C	1.51	1282.1
C(N) – H	1.09	1435.1
C – H	1.09	1435.1
	LEV	
1 – 6	1.22	3899.3
1 – 7	1.36	1746.7
7 – 9	0.98	2229.1
1 – 2	1.49	1261.6
2 – 3	1.51	1282.1
3 – 4	1.49	1261.6
4 – 5	1.49	1261.6
C – H	1.09	1435.1

# angles

angle	θ_{eq} / deg	k_θ / kJ mol^{-1} rad^{-2}
	$[N4444]^+$	
N – C(N) – C	106.5	710.0
C(N) – N – C(N)	112.2	519.1

N – C(N) – H	106.2	525.1
C – C(N) – H	110.5	383.0
C – C – H	110.5	383.0
C(N) – C – C	109.6	512.5
C – C – C	109.6	512.5
H – C(N) – H	108.8	310.7
H – C – H	108.8	310.7
**LEV**		
6 – 1 – 2	124.4	282.4
7 – 1 – 2	109.7	314.1
7 – 1 – 6	124.4	347.8
1 – 2 – 3	107.5	234.0
1 – 2 – H	108.4	195.7
3 – 2 – H	110.6	191.6
H – 2 – H	108.8	155.4
2 – 3 – 4	107.5	234.0
2 – 3 – H	110.6	191.5
H – 3 – 4	108.4	195.7
3 – 4 – 5	118.0	346.6
3 – 4 – 8	124.4	282.4
8 – 4 – 5	124.4	282.4
4 – 5 – H	108.4	195.7
H – 5 – H	108.8	155.4
1 – 7 – 9	112.0	175.6

# dihedrals

dihedral	δ / deg	k_ϕ / kJ mol^{-1}	m
**[N4444]$^+$**			
N – C(N) – C – C	0	-1.3556	1
	180	1.1506	2
	0	1.2343	3
N – C(N) – C – H	0	1.4477	1
	180	-1.1088	2
	0	0.5816	3
C(N) – N – C(N) – C	0	0.5230	3
C(N) – N – C(N) – H	0	0.5150	3
C(N) – C – C – C	0	0.2134	1
	180	1.4267	2
	0	0.6945	3
C(N) – C – C – H	0	1.3389	1
	180	-1.3180	2
	0	0.5523	3
C – C – C – C	0	0.2134	1
	180	1.4267	2
	0	0.6945	3
C – C – C – H	0	1.3389	1
	180	-1.3180	2
	0	0.5523	3
H – C(N) – C - H	0	0.5941	1
	180	-2.8995	2
	0	0.6569	3
H – C – C - H	0	0.5941	1

	180	-2.8995	2
	0	0.6569	3
**LEV**			
1 – 2 – 3 – 4	0	0.9288	1
	0	-2.3849	3
1 – 2 – 3 – H	0	-0.5356	1
	180	0.1213	2
2 – 1 – 7 – 9	0	-2.4393	1
	180	10.6232	2
	0	-1.1422	3
2 – 3 – 4 – 5	0	0.2134	1
	180	0.3682	2
	0	1.1422	3
2 – 3 – 4 - 8	0	1.7238	1
	180	0.2929	2
	0	0.6820	3
3 – 2 – 1 – 6	0	1.7238	1
	180	0.2929	2
	0	0.6820	3
3 – 2 – 1 – 7	0	-0.2469	1
	180	-0.6987	2
	0	0.4226	3
3 – 4 – 5 – H	0	-0.1506	1
	180	0.1799	2
	0	1.1129	3
4 – 3 – 2 – H	0	-0.5356	1
	180	0.1213	2
5 – 4 – 3 – H	0	-0.1506	1
	180	0.1799	2
	0	1.1129	3
6 – 1 – 2 – H	0	1.3807	1
	180	-2.9455	2
	0	0.6443	3
6 – 1 – 7 – 9	0	3.4769	1
	180	12.8700	2
	0	-0.1213	3
7 – 1 – 2 – 10	180	-1.3054	2
	0	0.6904	3
8 – 4 – 3 – H	0	1.3807	1
	180	-2.8455	2
	0	0.6443	3
8 – 4 – 5 – 14	0	1.3807	1
	180	-2.9455	2
	0	0.6443	3
H – 2 – 3 – H	0	0.5941	1
	180	-2.8995	2
	0	0.6569	3

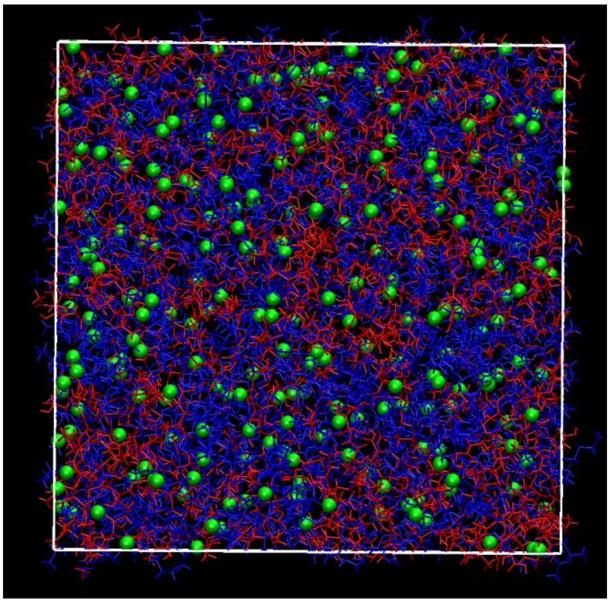

Figure S1. Example of cubic box used for periodic MD simulations for LEV : [N444]Cl (2 : 1) DES. Color code: (blue) $[N4444]^+$, (green) Cl^-, and (red) LEV. White lines show periodic boundary limits.

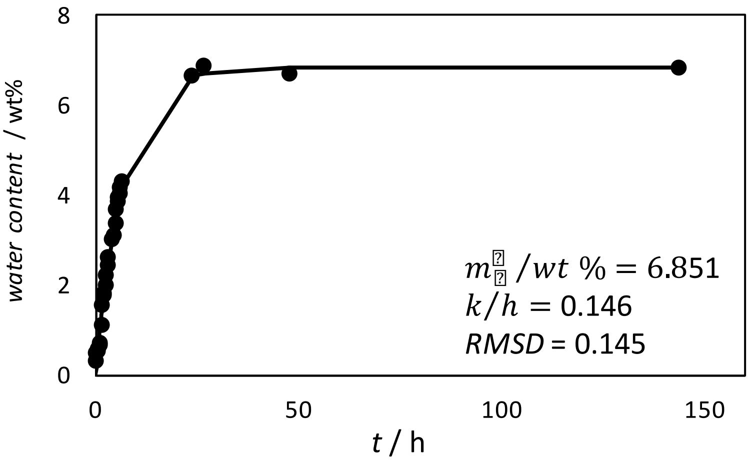

Figure S2. Kinetics for atmospheric water absorption in LEV : [N4444]Cl (2 : 1) DES at 298 K. Lines show fitting to the kinetic model, eq. (1), with the parameters reported at the bottom of the Figure.

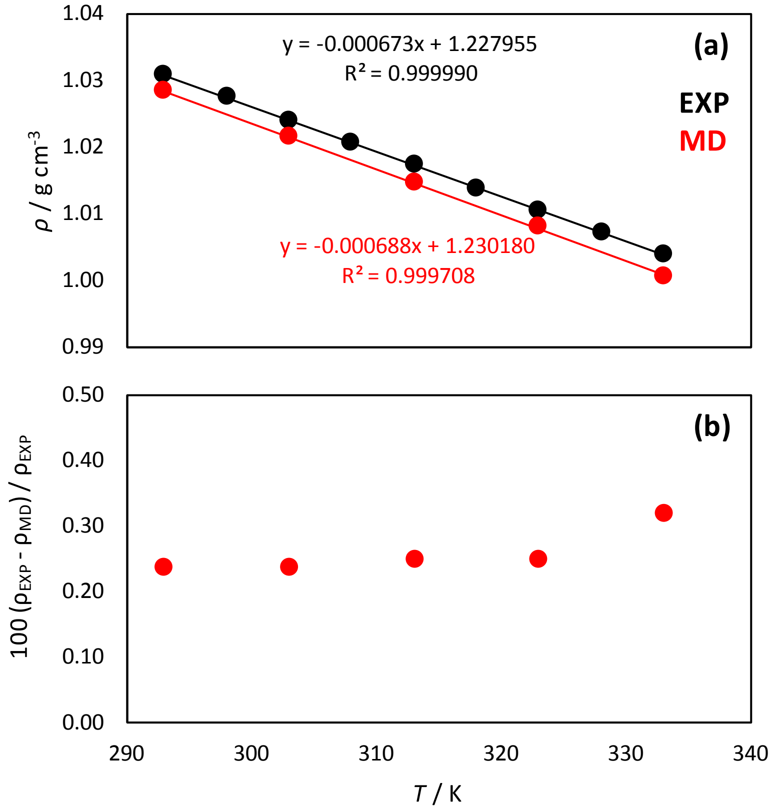

Figure S3. Comparison of density, ρ, from experimental (EXP, values from Table S2) and predicted molecular dynamics simulations (MD). In panel a, results of linear fits are reported.

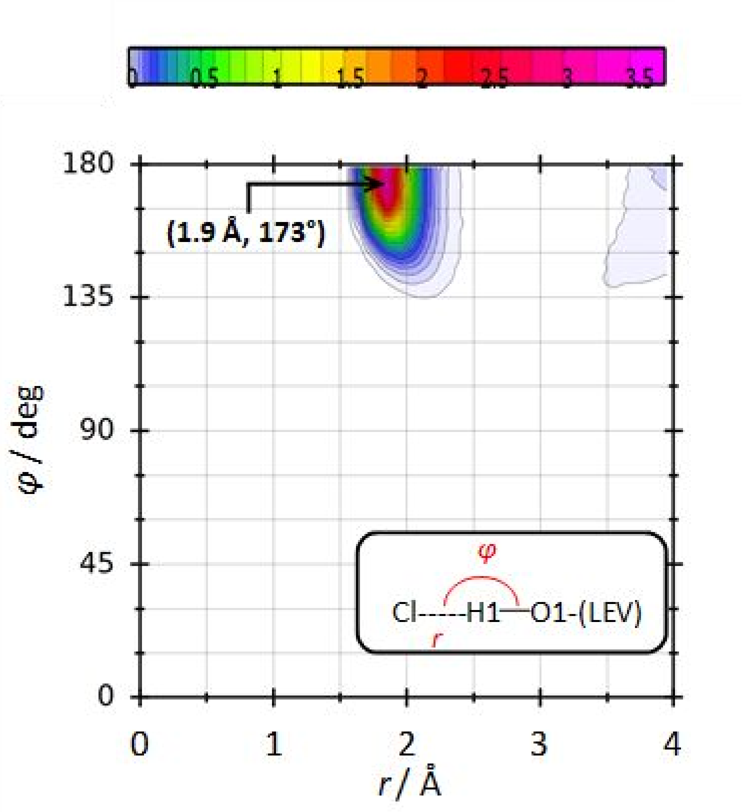

Figure S4. Combined distribution function for the reported distance and angle for LEV : [N4444]Cl (2 : 1) DES from MD simulations at 293 K and 1 bar. Atom labelling as in Figure 1. The arrow indicates the location of the maximum.
